# Optic Neuritis in an Adult Patient with Chickenpox

**DOI:** 10.1155/2012/371584

**Published:** 2012-12-19

**Authors:** Ana Rita Azevedo, Rita Simões, Filipe Silva, Susana Pina, Cristina Santos, Peter Pêgo, Filomena Silva, Susana Teixeira

**Affiliations:** ^1^Department of Ophthalmology, Hospital Prof. Doutor Fernando Fonseca EPE, 2720-276 Amadora, Portugal; ^2^Department of Neurology, Hospital Prof. Doutor Fernando Fonseca EPE, 2720-276 Amadora, Portugal; ^3^Retina Department, Hospital Prof. Doutor Fernando Fonseca EPE, 2720-276 Amadora, Portugal

## Abstract

Central nervous system involvement in a patient with primary infection with *Varicella zoster* virus is rare, especially in the immunocompetent adult. In particular, isolated optic neuritis has been described in a small number of cases. The authors present a case of optic neuritis in an immunocompetent patient. A 28-year-old woman presented to the emergency room with a history of headaches during the previous week, without visual symptoms. The examination was unremarkable, except for a rash suggestive of chickenpox and hyperemic and edematous optic disc, bilaterally. Visual acuity and neurological examination were normal. Two days later, she complained of pain on eye movement and decreased visual acuity, which was 20/32 in her right eye and 20/60 in her left eye. Four days after admission, her visual acuity started to improve, and two months later, she had 20/20 visual acuity in both eyes. To our knowledge, this is the first reported case of an immunocompetent adult in which a *Varicella zoster* virus associated optic neuritis presented with fundoscopic changes before decreased visual acuity. This suggests that this condition may be underdiagnosed in asymptomatic patients.

## 1. Introduction

*Varicella zoster* virus (VZV) causes two distinct clinical entities: chickenpox and zoster. Chickenpox is the result of primary infection and is more frequent in children. Reactivation of the virus is more common in adults after the sixth decade of life or in the immunosupressed patient. The most common complication of VZV primary infection is the secondary bacterial infection of cutaneous lesions. Central nervous system involvement is rare, especially in the immunocompetent adult [1]. In particular, isolated optic neuritis has been described in a small number of cases.

We report a case of an adult immunocompetent patient with optic neuritis in the context of primary infection with *Varicella zoster* virus.

## 2. Case Report

A 28-year-old caucasian woman presented to the emergency room with a history of a pressure-type frontal headache during the previous week; there was no exacerbation when lying down nor with Valsalva maneuvers and no accompanying features, namely, visual symptoms. Medical history was unremarkable except for venous thrombosis on her left leg 5 years before. She was not taking any drugs other than oral contraceptives. She had a son who was diagnosed with chickenpox two weeks before. She was febrile (temperature of 38°C), and there was a rash suggestive of chickenpox. Uncorrected visual acuity was 20/20 in both eyes, and the optic discs were swollen and hyperemic, with ophthalmological and neurological examinations otherwise normal. Two days later, she complained of ocular pain on eye movement and bilateral blurred vision. By that time, examination revealed reduced visual acuity of 20/32 in her right eye (OD) and 20/60 in her left eye (OS) and relative afferent pupillary defect on her left eye. She reported a difference in brightness of the red color, with less saturation in the left eye. 

Routine blood testing and immunological workup (ANA, ANCA, anticardiolipin antibodies, and complement levels) were normal. HIV serology was negative as well as VDRL, *Toxoplasma*, *Bartonella,* and *Borrelia *antibodies.

Cerebral computed tomography (CT) scan and magnetic resonance imaging (MRI) were normal. 

Cerebrospinal fluid (CSF) examination was unremarkable: clear fluid with a normal opening pressure of 130–140 mm H_2_O with no block when pressing the jugular vein, normal protein and glucose levels, less than 5 leukocytes, and no oligoclonal bands. Antibodies for the following microorganisms were negative in the CSF: Cytomegalovirus, Epstein Barr virus, Parvovirus, *Herpes simplex* virus, *Varicella zoster* virus, *Mycoplasma pneumoniae*, *Borrelia burgdorferi*, and *Leptospira *species. VDRL was also negative in the CSF.

**Figure 1:**
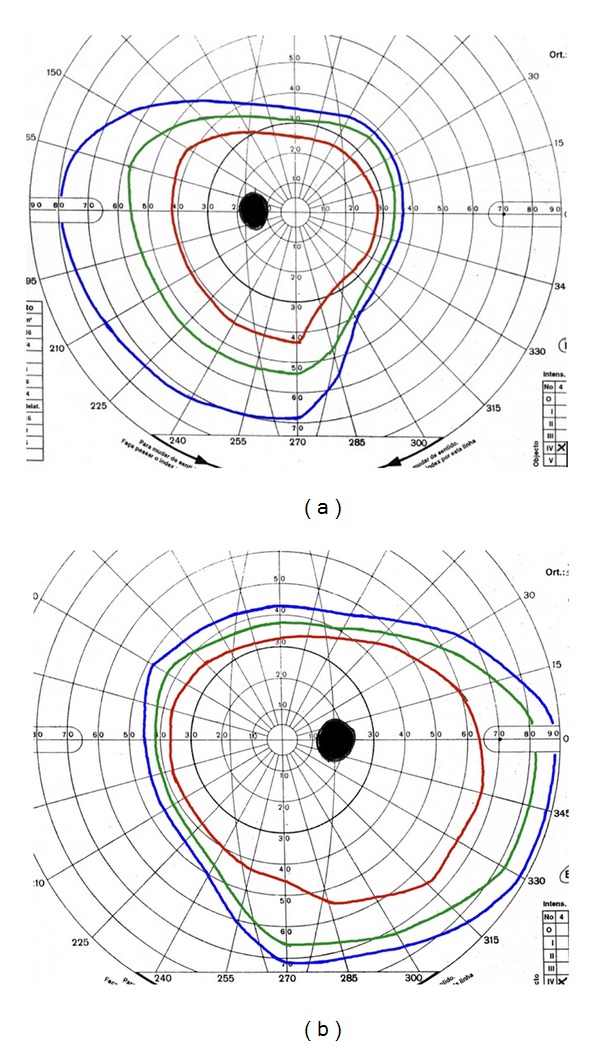
Goldmann perimetry left eye (a) and right eye (b) shows an enlarged blind spot more evident on the right eye and constriction of the superior and nasal fields especially in the left eye.

Goldmann perimetry showed an enlarged blind spot and constriction of the nasal field, especially in her left eye (Figure 1). Farnsworth Munsell 100 color vision test was abnormal in both eyes (in the red axis), with more pronounced defect in her left eye.

She was started on acyclovir 4 g/day *per os*, as is indicated to treat adult chickenpox.

**Figure 2:**
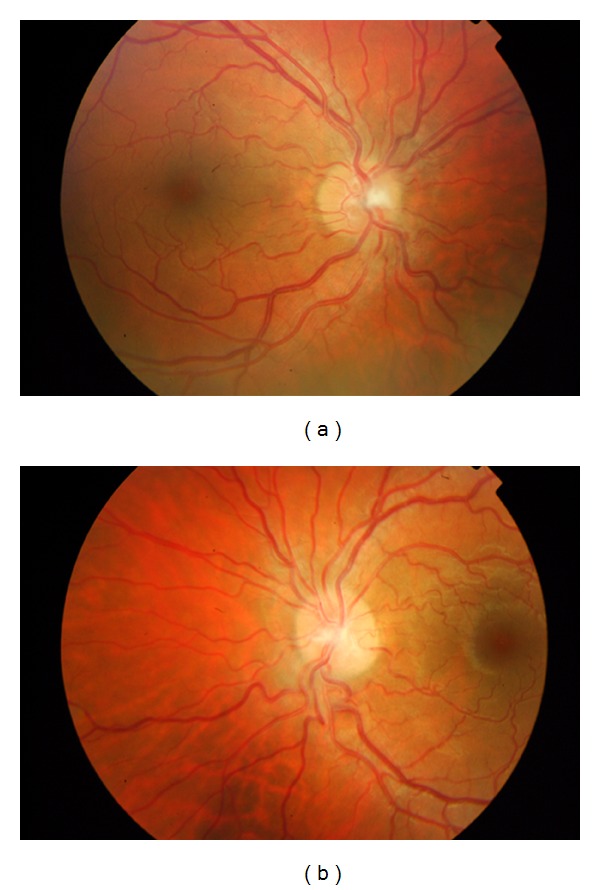
Retinal photographs of the right (a) and left (b) eyes 1 week after the initial visual symptoms. In the right optic disc, the limits are blurred more prominently in the superior and inferior poles. In the left eye optic disc, contour is almost normal.

Two days after the initial visual symptoms, visual acuity began to increase, and one week later, the optic disc edema had almost resolved (Figure 2). Visual acuity continued to improve, and two months later, she had a visual acuity of 20/20 bilaterally. Retinal fluorescein angiography one month after presentation was normal. She has been free of further attacks of optic neuritis for three years after the admission.

## 3. Discussion

The main differential diagnosis in the presence of a young woman with a previous history of venous thrombosis, taking oral contraceptives and presenting with headache and swollen optic discs, includes several causes of intracranial hypertension, especially cerebral venous thrombosis. However, the headache was not suggestive of intracranial hypertension, as there was no exacerbation when lying down or with Valsalva maneuvers. CT scan and gadolinium-enhanced MRI, with intracranial venous imaging, were normal, excluding venous thrombosis and parenchymal or meningeal lesions. Also, the CSF was normal, excluding meningeal inflammation and encephalitis, and there was a normal CSF opening pressure without block, excluding high intracranial pressure as the mechanism of the presenting signs and symptoms.

Optic neuritis may be the presenting feature of CNS demyelinating disorders. However, multiple sclerosis rarely presents with simultaneous, bilateral optic nerve involvement. As for neuromyelitis optica, although more frequently bilateral, retrobulbar neuritis is the rule, with a more severe course without complete recovery. Moreover, there were no white matter lesions in the MRI. These disorders did not seem to explain the clinical picture. 

The clinical diagnosis of chickenpox was supported by the presence of the characteristic vesicular rash and the close contact with her infected son some weeks before.

After excluding other etiologies of optic neuritis, the diagnosis of optic neuritis associated with *Varicella zoster* virus was made. Bilateral involvement, normal CSF (including absence of VZV antibodies), and the immunocompetent state of the patient suggested a postinfectious mechanism rather than a direct invasion of the nerve by the virus.

However, we would expect that, being an immune-mediated disease, the time between the rash and the decrease in the visual acuity would be longer than the observed in this patient. This could be explained by an earlier exposure to the virus, given that her son had been ill for more than 2 weeks before the patient initiated symptoms. Additionally, there are reports of neurologic disease without rash (*zoster sine herpete*) [2]. 

Previous reported cases of optic neuritis following chickenpox in immunocompetent adults had a similar course [3–5]. The decrease of visual acuity usually occurs between 2 to 38 days after the onset of the rash and is usually bilateral. Lee and colleagues, however, described a patient with a unilateral anterior optic neuritis that preceded the rash [6].

The pathogenesis of optic nerve involvement by the virus is not well understood. Some authors consider that there is direct nerve invasion in the case of reactivation of the latent infection and an immune-mediated lesion in the primary infection by the virus [7]. In fact, optic neuritis caused by reactivation of a VZV infection is frequently unilateral and associated with orbital inflammation, which suggests a distinct pathogenesis. On the other hand, VZV has not been isolated in the cerebral spinal fluid of immunocompetent patients [4]. This led some authors to hypothesize that the pathogenesis may differ with the state of patient's immunity. Galbussera et al. [4] suggested that in the immunocompetent, the process would be immune-mediated, and in the immunosuppressed, there would be direct viral invasion. In the former case a possible mechanism could be molecular mimicry between viral and neural antigens or incorporation of viral antigens into neural tissue such as cell membranes or myelin sheath in a genetically predisposed patient [3]. Considering this, some authors suggest the use of systemic corticosteroids in immunocompetent adults to accelerate recovery [3, 4]. Nevertheless, corticotherapy remains controversial since improvement of visual acuity not only occurs in its absence [4, 5, 8], but also despite its use, some patients maintain severe visual loss during follow up [3, 4]. Additionally, it may, theoretically, exacerbate the direct infection by the virus by diminishing the immune response, a phenomenon that is reported in HIV positive patients [9]. In our patient, we decided not to initiate corticosteroids, since visual acuity began spontaneous recovery 2 days after the beginning of visual symptoms.

This is a particularly interesting case because the decrease in visual acuity occurred 2 days after the optic disc changes were diagnosed, and, to our knowledge, this is the first case described with this presentation. In fact, in contrast to the other cases reported, our patient came to the emergency department early, before the appearance of visual symptoms. This finding helps to clarify the disease's natural history. Clinically, this is important because it suggests that one cannot completely exclude an optic neuritis in a patient with a swollen disc and normal visual acuity. This fact also suggests that optic neuritis due to VZV may be under diagnosed in patients that do not develop visual loss and so are not submitted to ocular fundus examination. In immunocompetent patients, this fact does not pose any problem; however, in immunosupressed ones, diagnosing a papillitis before symptoms arise may be important for the treatment and prognosis. Further studies are needed to clarify this issue.
